# Cognitive and Adaptive Effects of Early Growth Hormone Treatment in Prader–Willi Syndrome Patients: A Cohort Study

**DOI:** 10.3390/jcm11061592

**Published:** 2022-03-14

**Authors:** Aitana Ayet-Roger, Lorena Joga-Elvira, Assumpta Caixàs, Raquel Corripio

**Affiliations:** 1 Clinical and Health Psychology Department, Universitat Autònoma de Barcelona, 08193 Bellaterra, Spain; aitana.ayet@copc.cat; 2 Pediatric Neuropediatric Department, Parc Tauli Sabadell Hospital Universitari, 08208 Sabadell, Spain; 3 Institut d’Investigació i Innovació Parc Taulí (I3PT), Centre CERCA, 08208 Sabadell, Spain; acaixas@tauli.cat (A.C.); rcorripio@tauli.cat (R.C.); 4 Department of Medicine, Universitat Autònoma de Barcelona, 08208 Sabadell, Spain; 5 Endocrinology and Nutrition Department, Hospital Universitari Parc Taulí, 08208 Sabadell, Spain; 6 Pediatric Endocrine Department, Parc Tauli Sabadell Hospital Universitari, 08208 Sabadell, Spain

**Keywords:** Prader–Willi Syndrome, growth hormone treatment, cognition, adaptive behavior

## Abstract

Background: Prader–Willi Syndrome (PWS) is a genetically based neurodevelopmental disease characterized by obesity, hyperphagia, and mild to moderate intellectual disability. Treatment with growth hormone (GH) could provide cognitive benefits. The objective of the present study was to compare the cognitive and adaptive performance of 31 patients with genetically confirmed PWS grouped in two cohorts, one treated with GH before 2 years old (Group 1) and the other receiving the treatment later (Group 2). Method: We compared two variables necessary to diagnose intellectual disability: intellectual performance, using the Weschler scales, and adaptive behavior, using the DABS scale. The scores were analyzed by means of non-parametric statistical tests. Results: Group 1 (*n* = 10) obtained higher and statistically significant scores in Total Intelligence Quotient (TIQ), General Ability Index (GAI), and General Adaptive Behavior (GAB), implying better cognitive and adaptive performance compared to Group 2. Conclusions: Treatment with GH should be administered in the early stage of development (before 2 years old) to obtain greater benefits at the cognitive and adaptive levels.

## 1. Introduction

Prader–Willi syndrome is a genetic disease whose manifestations are caused by the lack of gene expression in the 15q11–q13 area of the allele of paternal origin. This condition can be due to three genetic causes: paternal deletion, maternal uniparental disomy, or mutation of the imprinting center of paternal origin [1]. It is a multisystemic and very heterogeneous syndrome that involves multiple symptoms: (1) physical: severe neonatal muscular hypotonia, a peculiar face, and a hypothalamic deficit, among which the growth hormone (GH) stands out; (2) behavioral: hyperphagia and insatiable food-seeking behavior, skin picking, ritualistic and obsessive compulsive behaviors, and emotional lability; (3) cognitive: intellectual disability, with an average intelligence quotient (IQ) between 55–60 and 70, and a verbal quotient usually below the manipulative quotient. Sufferers present attentional deficits, cognitive rigidity, and deficits in basic language skills [2,3], in addition to alterations in social cognition and characteristics similar to autism spectrum disorder (alterations in interpretation, facial and body expressiveness, interaction with peers, and development of theory of mind) [4]. The cognitive alterations presented aggravate the difficulties in understanding social cognition [5] and also underlie some of the characteristic behavioral alterations of the syndrome [6].

### 1.1. GH Treatment Effects

The administration of growth hormone (GH) is considered a cornerstone of the treatment of people with PWS due to its beneficial effects at a physical level, including growth stimulation and improvements in body composition, in particular, among others [7]. GH also has neurological effects in the development of the central nervous system, acting as a strong promoter of growth and differentiation. In fact, children with GH deficiency have shown reduced volume in structures such as the corpus callosum, hippocampus, thalamus, and basal ganglia, and this correlates with cognitive and motor function deficits [8]. GH action is also a necessary component of proper cerebellar development and adult function. This structure is implied in the coordination and regulation of muscular motor function, and in many cognitive functions.

There is controversy in the literature about the behavioral and psychological effects of GH treatment. Decreases in behavioral problems and increased vitality [9], reductions in depressive symptomatology [10], and improvements in well-being and quality of life [11,12] have been reported, although studies with longer follow-up periods have generally shown a neutral effect on behavior [13]. GH treatment is generally well tolerated—adverse effects are few, mostly mild, and do not usually require treatment discontinuation [12]. Cases of sudden death were reported after one year with GH treatment, and it was hypothesized that this was a possible side effect of the treatment. However, a recent review of the causes of death in patients with PWS found no difference between those who received treatment and those who did not. The authors found that the main cause of death in children with PWS is due to respiratory causes [14].

Some recent studies suggest that there are also effects at the cognitive level, with promising results so far.

### 1.2. GH Treatment and Cognition in Childhood

Longitudinal studies in children show that in the absence of GH treatment, they experience a deterioration in abstract verbal reasoning and visuospatial abilities. GH treatment prevents this deterioration, and increases in these abilities and in total IQ have been observed after 4 years of treatment [15]. These same results have been confirmed after 8 years of treatment, and a stable evolution of expressive verbal skills has also been observed [16].

Behavioral benefits appear earlier, as early as after 2 years of treatment [9], but behavior worsens after only 6 months of stopping treatment. In contrast, cognitive benefits do not appear until after 4 years [15], but remain stable until at least 1 year after GH treatment has ceased [17]. An interesting aspect is that after 8 years of treatment, up to 30% of patients who met the criteria for intellectual disability manage to achieve IQ figures >70, thus no longer meeting the cognitive criteria for the diagnosis of ID [16].

### 1.3. GH Treatment and Cognition in Adults

In adult patients treated with GH, improvements in work speed, cognitive flexibility, and motor performance have been described and are more significant after 18 months of treatment compared to 12 months. These skills allow better performance and efficiency when executing different tasks [18].

Even so, other reviews [19] conclude that there is no evidence of cognitive effects after GH treatment. It is worth mentioning that they include longitudinal studies with different durations, from 6 months to 4 years, and only one has a duration of more than 4 years of treatment. We believe that the unfavorable results are due to the fact that the effects do not appear until 4 years of treatment.

The aim of this study is to assess whether there is a benefit at the cognitive and adaptive levels after receiving early GH treatment (before two years of age) compared to treatment started later.

## 2. Materials and Methods

The study was approved by the Ethics Committee of the Consorci Corporació Sanitària Parc Taulí de Sabadell (CCSPT) (code 20215011). Voluntary participation was requested from all patients with PWS treated at the CSSPT and from members of the Catalan Association of PWS. Because the CCSPT is a reference center for PWS in Spain, patients not only from the corresponding health area but from all over the country are treated. Written informed consent was obtained from the parents and, where applicable, from the patients.

A retrospective observational empirical study was carried out in which two cohorts were compared, their main difference being having received, or not, treatment with GH before 2 years of age. Treatment with GH is part of the health care received by the vast majority of patients with PWS at our center, so assignment to one group or another depended on their previous treatment conditions.

### 2.1. Participants

The study began with 34 participants, who were chosen by convenience sampling. One patient was excluded due to having an IQ < 40, another because of the presence of comorbidity with cerebral palsy, and another due to lack of continuity in the study, leaving a final sample of 31 participants. All of them met the following inclusion criteria: (1) genetic confirmation of PWS; (2) IQ > 40 to be able to manage the cognitive battery; (3) Group 1 had received GH treatment since before 2 years old; (4) Group 2 had received GH treatment after 2 years old or had never received it.

Some Group 2 patients were receiving GH treatment for a maximum period of 6–12 months at the time of the study. However, given that the effects of GH on cognition do not occur until the fourth year of treatment [15,16], we considered that they were still valid to be included in the current study.

The following variables were recorded for all participants who received treatment: start date and duration of treatment, and socioeconomic level.

### 2.2. Instruments: Cognitive Functioning and Adaptive Behavior

Between one and two sessions were held with each participant to evaluate the study variables. The Weschler Intelligence Scales were used in their different versions to measure IQ according to age: WPPSI-IV for children up to 7:6 years old, WISC-V from 7:6 to 16:11 years old, and WAIS-IV for over 17:00 years. The results of the scales obtained were comparable to each other [17].

The cognitive indices presented in this study correspond to the names of the Weschler cognitive test indices: Total Intelligence Quotient (TIQ) was used as an intellectual global estimate, the General Ability Index (GAI) as an estimate of intellectual performance without dependence on executive functions, the CVI to assess verbal performance (made up of the *Similarities*, *Vocabulary,* and *Information* subtests, depending on the version used), and the *Symbol Search* or *Animal Search* subtest to assess processing speed (depending on the version administered). The DABS scale (Diagnostic Adaptive Behavior Scale) was administered for adaptive behavior, allowing for an estimation of the real performance of the patient in their environment and daily activities, and encompassing its three dimensions—conceptual, social, and practical skills—in addition to a general scale. It was answered by the parents or main caregiver, and we used the general scale of the test, the GAB.

The study was a unifactorial design with the independent variable having received treatment or not, and with the following dependent variables: Total Intelligence Quotient (TIQ), General Ability Index (GAI), Verbal Comprehension Index (CVI), processing speed (PS), and Global Adaptive Behavior (GAB).

### 2.3. Statistical Analysis

The statistical analysis was performed with SPSS Statistics for Windows software, version 25.0. For the descriptive analysis of the continuous variables, means and medians were used as descriptives of the central tendency, and the values located in the upper and lower limits of 95% confidence as measures of variability and dispersion. The dichotomous variables were presented with absolute value and percentage, *n* (%). For the analysis of differences, the Mann–Whitney U-test was used (comparison of differences between groups in the variables TIQ, GAI, CVI, PS, and CAG). The level of significance was set at 5%.

## 3. Results

### 3.1. Descriptive Data

We included 31 participants with PWS who met the aforementioned inclusion criteria. Ten participants received treatment with GH before 2 years old (Group 1) by prescription from their doctor and twenty-one participants did not receive treatment in the interval before 2 years old and so were placed in Group 2. Regarding socioeconomic demographics, 3.2% of the sample had a high socioeconomic level, 41.9% a medium-high level, 25.8% a medium level, 12.9% a medium-low level, and 16.1% a low level (Table 1).

The median age of Group 1 was 6 years, and the median age of Group 2 was 18 years (Table 2). Group 1 started treatment at a median age of 0.77 years, while Group 2 started at a median age of 4.41 years.

### 3.2. Cognition and Adaptive Behavior of the Total Simple

The participants’ total IQ ranged from 40 to 81, with a mean of 60.97 and a standard deviation of 12.30. The rest of the cognitive and adaptive values are shown in Table 3.

### 3.3. Comparison of Cognition and Adaptive Behavior in the Two Groups

The TIQ was 16 points higher in Group 1 than in Group 2 (median 74 vs. 58) (*p* = 0.009). The GAI was also higher in Group 1 than in Group 2 (median 77 vs. 62) (*p* = 0.043) (Table 4). No significant differences were found in verbal ability (76.5 vs. 67) (*p* = 0.186) (Table 4).

**Figure 1:**
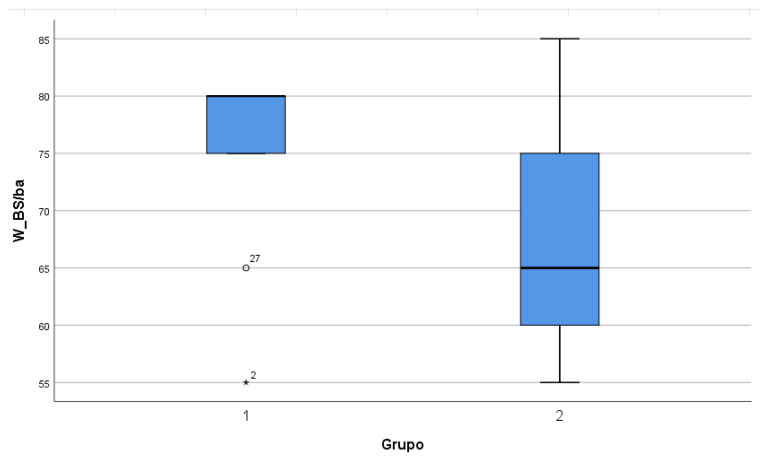
Boxplot of comparison of differences in processing speed for the two groups. (°) and (*): outlier values.

Regarding processing speed, Group 1 had a median score of 80 and Group 2 had a median score of 65. No statistically significant differences were found (*p* = 0.069), but a different trend was observed between the two groups, as can be seen in the comparison boxplot (Figure 1). Group 1 presented a very homogeneous distribution close to the median value, except for two outliers, which displaced the median value of the total group. On the other hand, Group 2 presented a much more dispersed distribution, and 75% of the cases had scores below almost the entire Group 1 (Table 4). Notably, comparison of the two groups in this variable was performed with a smaller sample (*n* = 9 in Group 1 and *n* = 19 in Group 2). This was because the three missing values were from outside the health area and there was a temporary limitation for visits, so the *Symbol Search* and *Animal Search* tests could not be performed. Regarding adaptive capacity, Group 1 obtained a higher median score than Group 2 (87.5 vs. 53) (*p* < 0.001) (Table 4).

## 4. Discussion

In the present investigation, the cognitive performance (TIQ, GAI, CVI, PS) and adaptive performance (GAB) of patients with PWS were evaluated, and their scores were compared according to whether they had received treatment with GH since before age 2 versus those who did not receive GH treatment until later or did not receive it at all. We found greater cognitive and adaptive performance in patients who had received early treatment. This is a pioneering study in Spain and, as far as we know, the first study to assess the cognitive effect of GH treatment started before the age of 2 years. Our group has previously shown that the treatment is safe in this age group [20,21]. Notably, the two groups compared are not equivalent in terms of the age variable. This is because currently, treatment is started earlier than in the past, so Group 1 included mostly younger patients. However, the tests carried out were adapted by age.

The length of treatment with GH of our Group 1 participants ranged from a minimum of 3.7 years to a maximum of 11.5 years. Some previous studies have found no global cognitive benefit after 2 years of treatment [9]; however, the benefits are evident after 4 years of treatment [15]. Taking this evidence into account, we considered that the time of treatment with GH in our sample was sufficient to explore the effects of treatment on cognitive capacity.

As in previous studies of cognition in patients with PWS [6,16], the distribution of the TIQ in the global sample studied was in the range of mild intellectual disability. However, when comparing the groups separately, it was found that Group 1 obtained a better performance in the TIQ, GAI, and GAB variables than Group 2. This finding is in favor of our initial hypothesis, which stated that early treatment with GH will lead to better global and adaptive cognitive capacity.

Similarly, Group 1 (early treatment) obtained an intellectual performance in the limit range of normality (TIQ of 74 and CAG of 87.5), while Group 2 (without early treatment) obtained a performance corresponding to an intellectual disability ranging from mild to moderate (TIQ of 58 and CAG of 53). This finding suggests that treatment with GH favors cognitive development, placing the TIQ and CAG figures in the limit range of normality instead of in the range of mild to moderate intellectual disability. These results are consistent with previous longitudinal studies in which, after administering GH treatment, researchers found that up to 30% of the participants increased their TIQ, moving from the range of mild disability to the range of borderline intellectual capacity [16]. As seen in the introduction, the findings can be related to the role that GH has in the development of different brain structures. There are also GH receptors in many brain structures, such as the cerebellum, that can be involved in many cognitive and motor functions [8].

Regarding the processing speed variable, a trend towards the limit of statistical significance was observed between the two groups, with the participants in Group 1 obtaining a better performance. The resulting lack of significance could be attributed to (1) the small sample size, inherent to studies on rare diseases and missing values on that variable, (2) and the presence of outliers in Group 1 that shifted the median value. This trend agrees with previous studies that have identified that treatment with GH favors an improvement in executive function and attention, decreased reaction time, and increased mental flexibility and motor function [18]. The differences found in PS could be related to benefits at the attentional level or the motor level. It would be interesting to investigate this by using a specific attention-only test and a specific motor coordination test; thus, it is not possible to separate both functions in the test used as PS.

The better performance found in the global cognition (TIQ and GAI) of our treated patients agrees with previous studies that have described increases in TIQ after 4 years of treatment with GH [15]. However, until now, only the effect of continued treatment has been assessed, regardless of the age at which it began and regardless of early treatment effects, which are important factors to take into account when examining the role of GH in brain development.

The findings of this study are especially important because currently, there are governmental/health limitations in relation to the age of initiation of GH treatment, sometimes resulting in very complicated administration at early ages and often starting above 2–4 years of age. These results contribute to the evidence that it is necessary to modify the existing limitations, thus favoring early treatment.

### Strengths and Limitations

One limitation of the study is that the sample is relatively small. However, since PWS is a rare disorder, with prevalence in Spain of 3.000, the sample of the present study can be considered a representative one.

GH treatment is a fundamental treatment for our patients with PWS and had already been prescribed by their doctors, so it was not possible to conduct an experimental study and deprive some patients of treatment to form an equivalent control group, due to ethical reasons. Nonetheless, the comparison of non-equivalent cohorts allowed us to compare the effects of early treatment to no early treatment, but it is worth mentioning that the lack of a matched group as a control makes it difficult to attribute all the differences found to the treatment. Therefore, more studies with larger samples are necessary, also taking into account other comorbidities that can impact cognitive function, such as metabolic status or thyroid function.

It would also be positive to evaluate not only global cognitive ability but also to study the profile of cognitive abilities in more detail. Further research including a more complete cognitive and motor assessment is needed.

Regarding strengths, this is the first study to consider adaptive functioning within the evaluation of “intellectual disability”, and thus to evaluate the effects of early treatment on cognition. It is also the first to study the cognitive effects of early GH treatment.

## 5. Conclusions

Group 1 obtained higher and statistically significant scores in Total Intelligence Quotient (TIQ), General Ability Index (GAI), and General Adaptive Behavior (GAB), implying better cognitive and adaptive performance for this group compared to the group without early treatment. The results suggest that early treatment with GH has beneficial effects on the cognitive and adaptive performance of patients with PWS and that it should be administered in the early stage of development (before 2 years) for greater benefits.

## Figures and Tables

**Table 1:** Descriptive data of the groups and the socioeconomic status of the 31 participants with Prader–Willi syndrome expressed as a frequency and percentage, *n* (%).

KERRYPNX	*n*	%
Group 1	10	32.3%
Group 2	21	67.7%
Socioeconomic status		
High	1	3.2%
Upper-middle	13	41.9%
Middle	8	25.8%
Low-middle	4	12.9%
Low	5	16.1%

Group 1 = early treatment; Group 2 = treatment after 2 years old or no treatment.

**Table 2:** Descriptive data of the 31 participants with Prader–Willi syndrome, with median and lower and upper limit values with 95% confidence interval, according to whether they belonged to Group 1 or Group 2. Expressed in years.

	Group 1		Group 2		*p*-Value
	Median	Lower and Limit Values	Median	Lower and Limit Values	
Age	6	(5.17–8.63)	18	(17.06–25.89)	<0.001 *
Age of starting treatment with GH	0.77	(0.76–1.25)	4.41	(3.33–11.79)	<0.001 *
GH treatment duration	5.90	(4.76–8.15)	12.33	(9.96–14.82)	0.001 *

Abbreviations: growth hormone, GH. Values denoted with (*) are significant.

**Table 3:** General descriptive data of the cognition and adaptive behavior of the entire sample, *n* = 31 (except for the PS variable, *n* = 28), expressed as the mean and standard deviation, m(sd), and minimum and maximum values.

	Mean (sd)	Minimum and Maximum Values
TIQ	60 (12.30)	40–81
GAI	66 (13.97)	40–94
VCI	70.65 (15.02)	45–98
PS	70 (9.71)	55–85
GAB	64.32 (20.95)	11–115

Abbreviations: Total Intelligence Quotient, TIQ; General Ability Index, GAI; Verbal Comprehension Index, VCI; processing speed, PS; Global Adaptive Behavior, GAB; standard deviation (sd).

**Table 4:** Cognitive and adaptive data according to belonging to Group 1 or 2, expressed as median values and lower and upper limits of 95% confidence. *p*-value for comparison of differences between the two groups using the Mann–Whitney U-test.

	Group 1		Group 2		*p*-Value
	Median	Lower and Upper Limits 95% of Confidence	Median	Lower and Upper Limits 95% of Confidence	
TIQ	74	(63.04–75.76)	58	(51.59–62.32)	*p* = 0.009 *
GAI	77	(67.50–80.10)	62	(56.91–70.52)	*p* = 0.043 *
CVI	76.5	(67.77–82.43)	67	(60.95–76.10)	*p* = 0.186
PS	80	(68.07–81.93)	65	(63.13–72.13)	*p* = 0.068
GAB	87.5	(70.49–97.11)	53	(48.25–61.84	*p* < 0.001 *

Abbreviations: Total Intelligence Quotient, TIQ; General Ability Index, GAI; Verbal Comprehension Index, VCI; processing speed, PS; Global Adaptive Behavior, GAB. Values denoted with (*) are significant.

## Data Availability

Data are available under request.
